# Cultural conceptions of self and their impact on the clinical expression and management of identity disturbances in schizophrenia: a philosophical and psychiatric inquiry in Arab-Muslim populations

**DOI:** 10.1192/j.eurpsy.2026.11886

**Published:** 2026-07-31

**Authors:** F. Zaouali, I. Anes

**Affiliations:** 1 Emergency department and mobile emergency and resuscitation service, Tahar Sfar University Hospital, Mahdia; 2 Outpatient psychiatric consultation, Hadj Ali Soua Regional Hospital, Ksar Hellal, Monastir, Tunisia

## Abstract

**Introduction:**

The experience and expression of identity disturbances in schizophrenia are profoundly influenced by cultural conceptions of the self. In Arab-Muslim societies, traditional philosophical and religious views shape patients’understanding of selfhood, potentially modifying symptom presentation and treatment engagement.

**Objectives:**

This study aims to investigate how cultural and philosophical constructs of the self within Arab-Muslim contexts affect the clinical manifestation and management of identity disturbances in schizophrenia.

**Methods:**

A mixed-methods approach was employed. First, a systematic literature review (2000–2025) was conducted across PubMed, PsycINFO, Scopus, and Google Scholar using keywords: “schizophrenia,” “identity disturbances,” “self-concept,” “Arab-Muslim,” “culture,” and “philosophy.” Inclusion criteria encompassed studies addressing cultural or philosophical aspects of self in schizophrenia. Secondly, qualitative interviews were conducted with 20 Arab-Muslim patients diagnosed with schizophrenia at a regional psychiatric clinic, exploring their subjective experiences of self and illness. Data were analyzed using thematic analysis to identify culturally specific patterns influencing symptom expression and treatment adherence.

**Results:**

The systematic review identified 18 relevant studies, with 65% reporting that cultural and spiritual beliefs significantly shape symptom expression in schizophrenia among Arab-Muslim populations. Qualitative interviews with 20 patients revealed that 70% interpreted their identity disturbances through spiritual frameworks, such as possession or divine punishment. Additionally, 60% reported initial reliance on religious or traditional healers before seeking psychiatric care. Treatment adherence was affected, with 45% of patients expressing skepticism towards biomedical interventions when cultural beliefs were not acknowledged. Family influence was prominent, with 80% of participants noting that familial attitudes guided help-seeking behavior and symptom The literature and patient narratives revealed that Arab-Muslim cultural frameworks emphasize a relational and spiritual conception of self, contrasting with Western individualistic models. This influences the presentation of identity disturbances, often manifested through spiritual interpretations.

**Conclusions:**

Cultural conceptions of self significantly shape identity disturbances in schizophrenia among Arab-Muslim populations. Incorporating philosophical and cultural insights into psychiatric practice enhances diagnostic accuracy and fosters culturally congruent, patient-centered care. Future research should focus on developing culturally adapted clinical tools and therapeutic interventions to improve outcomes in this population.

**Disclosure of Interest:**

None Declared

